# Oncofoetal fibronectin – a tumour-specific marker in detecting minimal residual disease in differentiated thyroid carcinoma

**DOI:** 10.1038/sj.bjc.6602741

**Published:** 2005-08-09

**Authors:** E Hesse, P B Musholt, E Potter, T Petrich, M Wehmeier, R von Wasielewski, R Lichtinghagen, T J Musholt

**Affiliations:** 1 Clinical Chemistry, Hannover University Medical School, Carl Neuberg Str. 1, D-30625 Hannover, Germany; 2 Nuclear Medicine, Hannover University Medical School, Carl Neuberg Str. 1, D-30625 Hannover, Germany; 3 Pathology, Hannover University Medical School, Carl Neuberg Str. 1, D-30625 Hannover, Germany; 4 General & Abdominal Surgery, Gutenberg University Mainz, Langenbeck Str. 1, 55101 Mainz, Germany

**Keywords:** thyroid cancer, thyroglobulin mRNA, oncofoetal fibronectin mRNA, minimal residual disease, real-time RT–PCR, molecular diagnostics

## Abstract

Supposedly, thyrocyte-specific transcripts such as thyroglobulin (Tg) and thyroid-stimulating hormone receptor (TSH-R) were proposed to be useful for the diagnosis of circulating tumour cells in patients suffering from differentiated thyroid carcinoma (DTC). However, several research groups reported blood-borne Tg transcripts in healthy individuals. This study determines in particular the origin of Tg mRNA in nucleated blood cells and analyses whether other tumour-associated sequences are absent in leukocytes, but widely expressed in DTC. Therefore, expression analyses for Tg, TSH-R, cytokeratin 19 (CK 19), human telomerase reverse transcriptase (hTERT) and oncofoetal fibronectin (onfFN) were carried out using cDNAs derived from (1) leukocyte fractions, (2) 18 follicular thyroid carcinomas (FTCs) and 48 papillary thyroid carcinomas (PTCs), and (3) leukocytes of two thyrocyte-depleted individuals treated for C-cell carcinoma of the thyroid. Expression of onfFN was additionally analysed by semiquantitative RT–PCR and by quantitative fluorescence-based real-time PCR. Tg and TSH-R expression was demonstrated not only in both athyroid individuals, but in all leukocyte subgroups tested, while hTERT was absent in resting CD4^+^ cells and only weakly expressed in the CD8^+^ group. CK 19 was notable in each leukocyte population except for resting CD14^+^, as well as for activated and resting CD19^+^ cells. All blood cell fractions proved negative for onfFN mRNA, whereas its presence in thyroid carcinoma was 78/98% (FTC/PTC). Threshold cycle values were calculated at: porphobilinogen deaminase (PBGD) =25.95±0.73 (FTC)/24.55±5.43 (PTC) (*P*=0.2878); onfFN=25.48±3.15 (FTC)/21.44±3.44 (PTC) (^*^P=0.0001). Finally, onfFN transcripts were detected in blood samples of six out of nine patients with known DTC metastases, demonstrating a reliable assay functionality. We propose that real-time RT–PCR of onfFN mRNA is superior to other markers in monitoring minimal residual disease in DTC with regard to both assay sensitivity and specificity.

Differentiated thyroid carcinoma (DTC) appears with an incidence of approximately three out of 100 000 and ranks 14th among the major malignancies (Mazzaferri, 2000). In Europe and North America, DTC is the most common endocrine malignancy accounting for 1% of all tumours (Parker *et al*, 1997). Periodic radioiodine scanning and serum thyroglobulin (Tg) measurement are an integral part of the postoperative care. However, in about 13% of a population, interfering anti-Tg antibodies are present (Pedersen *et al*, 2003) which may lead to a decreased Tg immunoassay sensitivity (Ringel *et al*, 1998; Spencer *et al*, 1998). Additionally, appropriate serum Tg measurement requires a TSH stimulus achieved by rhTSH administration, which obviates the need for long-term thyroid hormone withdrawal, leading to patient morbidity due to symptomatic hypothyroidism. Both concepts may cause tumour growth stimulation. Despite improved therapy concepts and excellent prognosis, tumour relapse and metastases do occur in 10% of patients (Mazzaferri, 2000). Thus, over the last decade, many studies have attempted to improve DTC diagnosis and patient follow-up using RT–PCR-based approaches in order to track down circulating tumour cells (Ditkoff *et al*, 1996; Tallini *et al*, 1998; Ringel *et al*, 1999; Wingo *et al*, 1999; Biscolla *et al*, 2000; Bojunga *et al*, 2000; Weber *et al*, 2000; Bugalho *et al*, 2001; Takano *et al*, 2001; Eszlinger *et al*, 2002; Span *et al*, 2003; Chinnappa *et al*, 2004; Elisei *et al*, 2004; Li *et al*, 2004).

Thyroglobulin mRNA was thought to originate from circulating thyrocytes (Ringel *et al*, 1998), but other studies demonstrated that physiological blood cells such as leukocytes are capable of ectopic Tg mRNA transcription (Tallini *et al*, 1998; Bojunga *et al*, 2000; Bugalho *et al*, 2001; Takano *et al*, 2001; Eszlinger *et al*, 2002). To overcome these inherent limitations, one must consider either cumbersome tumour cell enrichment from peripheral blood or amplification of tumour-specific markers. However, the exact origin of Tg transcripts in healthy volunteers remains to be elucidated, and molecular approaches should integrate sequences lacking the phenomenon of illegitimate transcription in order to obtain signals specific for the respective malignant cell.

In order to monitor patients with DTC for minimal residual disease by blood assays, we decided to analyse promising candidate transcripts, which have been discussed to be useful in this context. For instance, cytokeratin 19 (CK 19) transcripts were used in several studies as a molecular marker for tumour cell detection (Datta *et al*, 1994; Burchill *et al*, 1995; Eltahir *et al*, 1998; Trummer *et al*, 2000).

Furthermore, human telomerase reverse transcriptase (hTERT) activity is associated with the majority of malignancies, and its activity is absent in most benign tissues (Kim *et al*, 1994; de Kok *et al*, 1999; Holt and Shay, 1999; Soria *et al*, 1999).

During tumorigenesis, fibronectin is alternatively spliced, leading to isoforms such as the extracellular domains A/B (ED-A/ED-B) and the III-connecting segment (III-cs) (Takano *et al*, 1997, 1999, 2000). These uniquely glycosylated isoforms are predominantly expressed by foetal and neoplastic cells, and have been designated oncofoetal fibronectin (onfFN) (Guller *et al*, 2003).

The overall purpose of this study is to analyse the expression of Tg, thyroid-stimulating hormone receptor (TSH-R), CK 19, hTERT, and onfFN transcripts in leukocytes, as well as in papillary thyroid carcinomas (PTCs) and follicular thyroid carcinomas (FTCs), respectively. Conspicuous mRNA sequence abundance in DTC combined with unequivocal absence in leukocytes will serve as a valuable diagnostic tool in cancer cell detection in the peripheral blood, which is finally tested in a small group of patients. Herein, we intentionally confine our focus to a selected number of mRNA transcripts which can be useful in future investigations of minimal residual disease in DTC with promising clinical applications.

## MATERIALS AND METHODS

### Patient samples and blood

The local university ethics committee approved the study and informed consent was obtained from all patients. Tissue samples from patients suffering from FTC or PTC and from nonmalignant thyroid glands were collected intraoperatively (FTC: *n*=18, PTC: *n*=48, nonmalignant thyroid=4), immediately snap-frozen in liquid nitrogen, and stored at −80°C. Of the FTC group, five tumours represented oxyphilic thyroid carcinomas displaying follicular architecture in more than 30% of the tumour (Musholt *et al*, 2003). The diagnosis of all thyroid tissues was histopathologically confirmed by an experienced pathologist prior to analysis.

Commercial cDNA from different leukocyte fractions was used as template for PCR (MTC Panel, Clontech, Heidelberg, Germany).

Two individuals with a history of medullary thyroid carcinoma (MTC=C-cell carcinoma) who additionally underwent radio-iodine treatment following total thyroidectomy served as negative controls for thyrocyte-depleted human blood. For onfFN expression analysis, blood samples from nine patients suffering from either FTC or PTC metastatic disease and from eight healthy control individuals were obtained. Mononuclear peripheral blood cells were isolated from 5 ml EDTA treated blood by Ficoll-Paque® (Nycomed, Oslo, Norway) density gradient centrifugation according to the manufacturer's protocol.

### RNA extraction

Total RNA was extracted from tissue samples (20 mg) by TRIzol Reagent (Invitrogen, Karlsruhe, Germany), and genomic DNA was eliminated by DNase I digest (Roche Diagnostics, Mannheim, Germany) prior to RNA purification (RNeasy Kit, Qiagen, Hilden, Germany). Peripheral blood-derived total RNA was isolated using only the column-based RNeasy Kit (Qiagen). The total RNA concentration was measured fluorometrically by use of RiboGreen RNA Quantitation Kit (MoBiTec, Goettingen, Germany).

### RT–PCR

Synthesis of cDNA was performed using either 4 *μ*g (tissue samples) or 1 *μ*g (blood samples) of total RNA and 3 *μ*l oligo-dT_20_ primer (25 pmol *μ*l^−1^) (MWG, Ebersberg, Germany). Following incubation at 70°C for 10 min, a reaction mixture containing 4 *μ*l dNTPs (5 mmol *μ*l^−1^ of each dNTP) (Roche Diagnostics), 8 *μ*l 5 × SuperScript II Buffer, 4 *μ*l DTT (0.1 mmol *μ*l^−1^) and 1 *μ*l SuperScript II (200 U *μ*l^−1^) (Invitrogen) was added and then incubated for another 50/10 min at 42/70°C. The final volume of this mixture was adjusted to 100 *μ*l using tRNA solution (0.25 mg ml^−1^) (Roche Diagnostics). For the PCR reaction, 9.5 *μ*l of diethyl pyrocarbonate (DEPC)-treated double-distilled H_2_O (ddH_2_O) was added to 2.5 *μ*l of cDNA. Amplification was performed in 25 *μ*l total volume containing 2.5 *μ*l 10 × reaction buffer (Promega, Mannheim, Germany), 1.5 *μ*l MgCl_2_ (25 mmol *μ*l^−1^), 0.4 *μ*l Taq polymerase (5 U *μ*l^−1^) (Qiagen), 1 *μ*l DTT (25 mmol *μ*l^−1^), 0.5 *μ*l of each oligonucleotide (50 pmol *μ*l^−1^) (MWG), 1 *μ*l dNTPs (5 mmol *μ*l^−1^) (Roche Diagnostics) and 13 *μ*l DEPC-ddH_2_O. After incubation at 94°C for 3 min, a touch-down PCR programme was started at 94°C/annealing temperature +10/72°C for 30/45/60 s with increments of 1°C each for two cycles, followed by 15 cycles at the specific annealing temperature and an incubation at 70°C for 5 min. Reaction mixtures either lacking template or containing RNA instead of cDNA served as negative controls. The primer sequences and annealing temperatures used in this study are listed in Table 1.

### Polyacrylamide (15%) gel electrophoresis (PAGE)

Vertical PAGE was used for PCR product separation, and the amplicons were visualised by silver staining as described previously (Lichtinghagen *et al*, 1994). Resulting signals were subdivided semiquantitatively in the categories high, intermediate, low and none according to their band intensities.

### Real-time PCR

Fluorescence resonance energy transfer (FRET) technology (LightCycler, Roche Diagnostics) was used for real-time PCR analysis. Expression of the low-abundance housekeeping gene PBGD served as an internal positive control in each assay performed. A reaction mixture contained 2.5 *μ*l cDNA, 2 *μ*l PBGD/onfFN primer (10 pmol *μ*l^−1^) (see Table 1), 2 *μ*l FRET probes (4 pmol *μ*l^−1^) PBGD-FITC (tcctcctggcttcaccatcggagc-FITC), PBGD Cy5.5 (Cy5.5-tctgcaagcgggaaaaccctcatg), onfFN-IIICS-FITC (ggtatgacactggaaatggtattcagct-FITC), onfFN-IIICS- Cy5.5 (Cy5.5-tggcacttctggtcagcaacccag) (MWG), 2 *μ*l Fast Start DNA Master Hybridization Mix, 3.2 *μ*l MgCl_2_ (25 mmol l^−1^) and 1 *μ*l DMSO (Roche Diagnostics). The mixture was adjusted to 20 *μ*l using DEPC-ddH_2_O. For denaturation, a temperature of 95°C was maintained for 12 min. Amplification was performed at 95/59/72°C for 15/12/10 s (PBGD: 50 cycles, onfFN: 38 cycles, slope: 20°C s^−1^) without colour compensation mode. A standard calibration curve was constructed for each sequence after cloning of positive controls as described previously (Lichtinghagen *et al*, 1995), and the assay sensitivity was determined using dilutions of the positive controls. Reaction mixtures either lacking template or containing RNA served as negative controls. In order to determine the real-time PCR sensitivity, an absolute quantification of PBGD and onfFN target sequences was calibrated by use of cloned cDNA serial dilutions. The standard curve started at 10^8^ (onfFN)/10^7^ (PBGD) initial cDNA copies. After measurement of the relative fluorescence intensity (channel F3/F1) for each sample, a proportional baseline adjustment for sample quantification was carried out by second derivative maximum calculation, and the amount of each mRNA transcript was expressed as a threshold cycle (ct) value.

Resulting ct values were used for statistical analysis to determine the significant differences of onfFN and PBGD mRNA expression in PTCs and FTCs, respectively, using a two-sample *t*-test.

## RESULTS

### Illegitimate transcription of supposedly thyrocyte-specific transcripts in leukocytes

Activated and resting leukocyte subgroups were analysed for transcription patterns of thyroid-associated mRNA products by RT–PCR analysis. Whereas each leukocyte subgroup tested positive for transcripts of Tg, TSH-R, and PBGD, divergent expression profiles of hTERT, CK 19, and onfFN were observed in CD subgroups.

**Figure 1:**
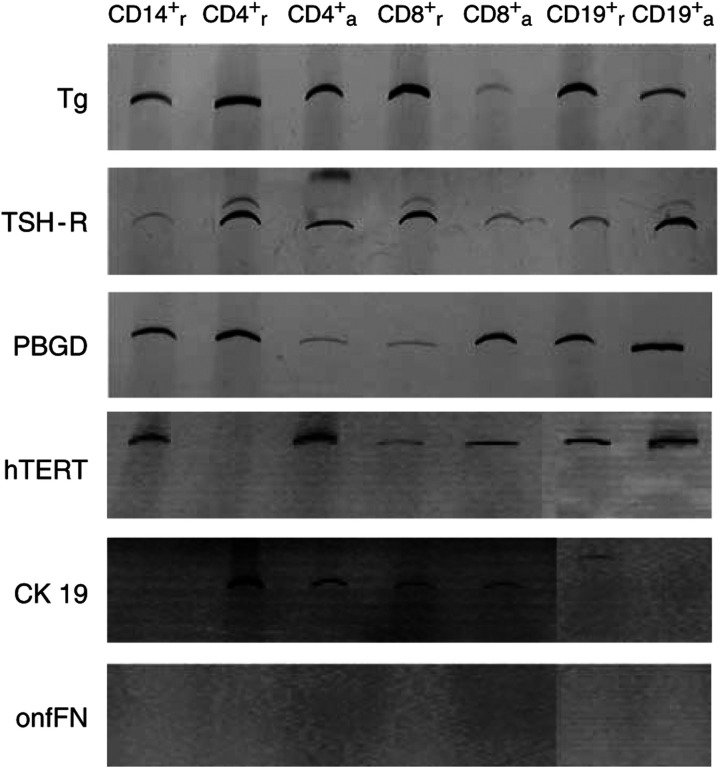
Tg, TSH-R, the low-abundance house-keeping gene PBGD, hTERT, CK 19, and onfFN mRNA transcripts were PCR-amplified using cDNA from different leukocyte subgroups. Amplicons were visualised by PAGE, followed by silver staining. The subscripts (r and a) indicate resting or activated status, respectively, for each subgroup.

hTERT transcripts were not detected within resting CD4^+^ cells and were found at only low levels within the CD8^+^ population. Resting CD14^+^ cells as well as activated and resting CD19^+^ cells lacked CK 19 transcripts. All other fractions expressed CK 19. The weakest expression was demonstrated in the CD8^+^ group. No leukocyte subgroup tested transcribed onfFN mRNA (Figure 1).

**Figure 2:**
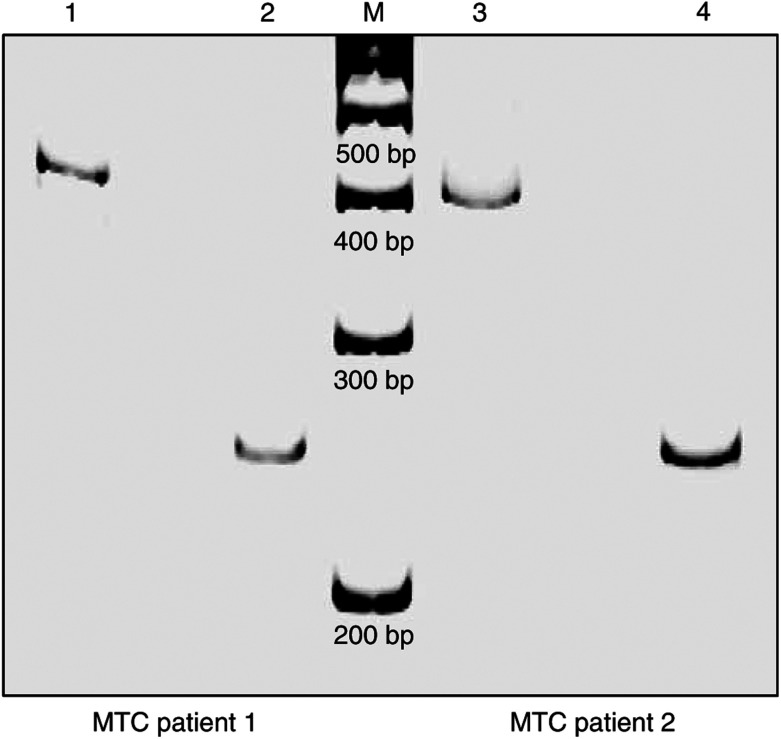
In the study of minimal residual disease in DTC, two thyrocyte-depleted individuals – suffering from medullary thyroid carcinoma and treated with thyroidectomy as well as with radio-iodine therapy – served as negative controls (MTC patients 1 and 2). RT–PCR amplification for Tg (lanes 1+3, product size: 405 bp) and PBGD (lanes 2+4, product size: 253 bp) transcripts was carried out following harvest of nucleated peripheral blood cells by density gradient centrifugation (M=100 bp DNA ladder). A notable expression of the supposedly thyrocyte-specific Tg is shown (PAGE, silver staining).

Nucleated peripheral blood cells of two patients without any residual thyroid tissue following treatment for medullary thyroid carcinoma were positive for Tg mRNA (Figure 2).

### Tissue samples

**Figure 3:**
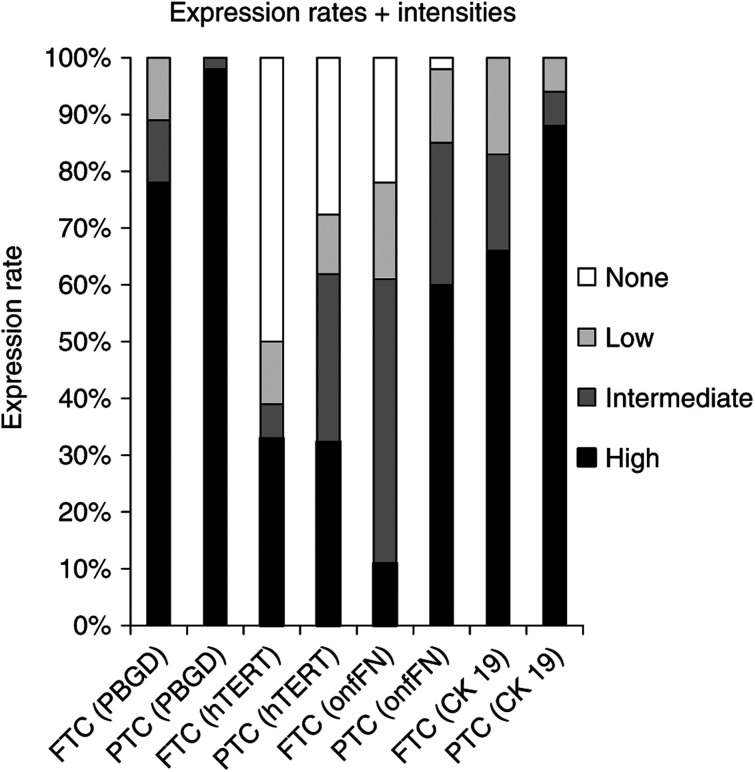
Tissue samples of 18 FTCs and 48 PTCs were subjected to RT–PCR-based semiquantitative expression analysis for PBGD, hTERT, onfFN, and CK 19, respectively. Following PAGE and silver staining, band intensities were estimated and illustrated.

Total RNA was extracted from 48 PTCs, 13 FTCs, and five oxyphilic follicular thyroid carcinomas (oxyFTCs) – often synonymously designated as Huerthle cell carcinomas – and four nonmalignant thyroid tissue samples. RT–PCR for PBGD, hTERT, onfFN, and CK 19 was performed and analysed by PAGE. As expected, PBGD expression was detectable in all tumours examined. Half of the FTC samples and 29% of the PTC tumours were negative for hTERT. High levels of hTERT were observed in 34/33% (FTC/PTC), intermediate levels in 6/31%, and a low level of expression occurred in 11% of each tumour group. Detectable levels of onfFN transcripts were present in 78/98% (FTC/PTC). The FTC samples revealed high expression in 11% of the tumours, whereas a high expression of onfFN was observed in 60% of PTCs. Intermediate and low levels (17%) of onfFN were detected in one-half of the FTC samples. A quarter of all PTC tumours displayed intermediate and low (13%) onfFN signals. All tumour tissues transcribed CK 19 at a relatively high level (66% for FTCs; 88% for PTCs). Intermediate and low levels were measured in 17% of FTCs and 6% of PTCs examined (Figure 3). There was no specific expression pattern detectable discerning oxyFTCs from FTCs without oxyphilic histomorphology. Nonmalignant thyroid tissue samples were only analysed for onfFN mRNA expression and were found to be negative.

### Quantification

In order to determine the real-time PCR sensitivity, an absolute quantification of PBGD and onfFN target sequences was calibrated by use of cloned cDNA serial dilutions. Sensitivity of the assay was determined to be 10^3^ (onfFN)/10^4^ (PBGD) cDNA molecules per reaction. The correlation coefficient was *R*=0.99 for onfFN as well as for PBGD.

In order to determine the inter-assay precision, two samples of both onfFN and PBGD standards of different concentrations were cycled eight times in a row. Amplification of 10^6^ cDNA copies (onfFN and PBGD) revealed a mean ct value of 19.75 (s.d.±0.16, CV=0.8%) for onfFN, and 22.08 (s.d.±0.56, CV=9.2%) for PBGD, respectively. Amplification of a lower concentration (onfFN: 10^3^, PBGD: 10^5^) proved a mean ct value of 25.84 (s.d.±2.39, CV=9.2%) for onfFN and 24.9 (s.d.±0.22, CV=0.9%) for PBGD.

**Figure 4:**
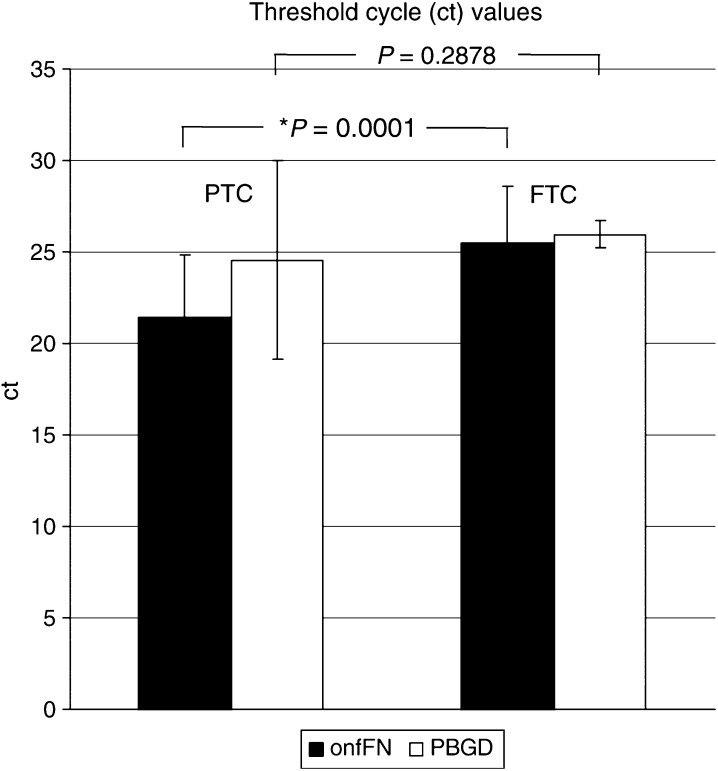
Results of a real-time PCR quantification of PBGD and onfFN transcripts performed on cDNA from 18 FTC and 48 PTC samples are given using ct values after second derivative maximum analysis.

PBGD and onfFN transcripts of 18 FTC and 48 PTC samples were then quantified by real-time PCR using the calibration curve made of diluted cloned controls. Calculation was performed by second derivative maximum analysis and the quantity of each transcript was expressed as ct values. A mean PBGD ct was observed at 25.95±0.73 for FTC samples and 24.55±5.43 for PTC tumours, demonstrating no significant difference (*P*=0.2878). onfFN ct were calculated at 25.48±3.15 and 21.44±3.44 for FTC and PTC samples, respectively. The expression difference between both tumour groups was significant (^*^P=0.0001) (Figure 4).

**Figure 5:**
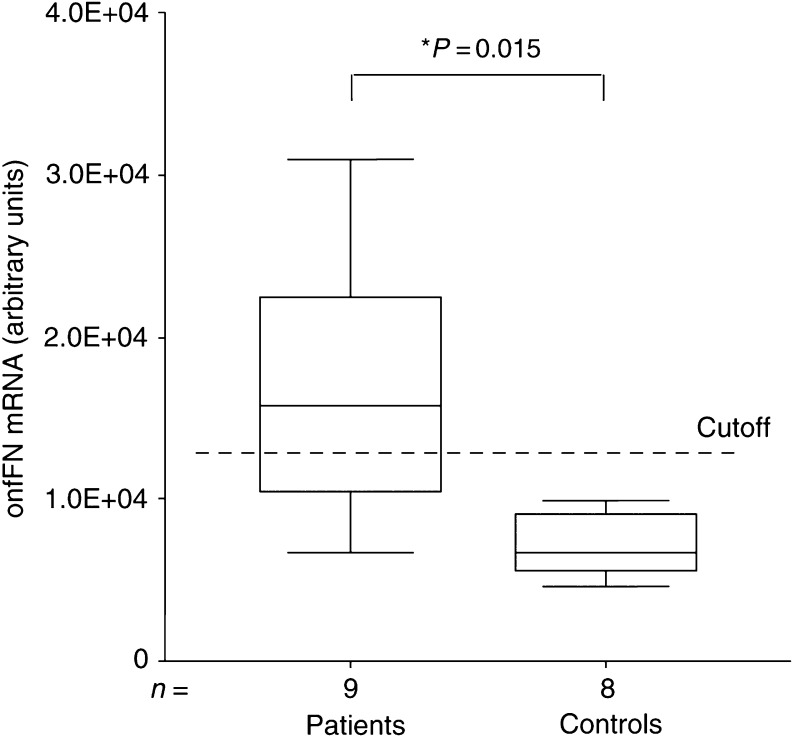
Quantitative real-time PCR data are shown in a box plot graph. Based on an unspecific background signal obtained in healthy controls (*n*=8), we calculated a cutoff value (3 × s.d.+mean). onfFN transcripts were detected in the peripheral blood in 67% of patients (*n*=9) suffering from DTC metastatic disease. Expression rates of both groups are presented in arbitrary units based on copy numbers of a recombinant onfFN standard, leading to a better inter-assay precision in daily routine diagnostic.

Peripheral blood from patients with confirmed FTC or PTC metastases tested positive for onfFN mRNA expression in six out of nine cases using the established assay. The signal was significantly (^*^P=0.015) higher in patients (mean: 1.7 × 10^4^, s.d.: 8.6 × 10^3^ (arbitrary units)) compared to controls (*n*=8) (mean: 7.1 × 10^3^, s.d.: 2.0 × 10^3^ (arbitrary units)). A cutoff value (3 × s.d.+mean=1.3 × 10^4^ (arbitrary units)) was applied to identify positive samples (Figure 5). The results were confirmed three times with a high reproducibility.

Using a thyroid carcinoma cell line dilution model, we found one tumour cell in 10^6^ mononuclear cells (data not shown).

## DISCUSSION

In the recent past years, a considerable number of papers reporting tissue-specific or cell-specific gene expression analyses for diagnosis of minimal residual disease in DTCs following total thyroidectomy were published. Some laboratories focused on Tg mRNA as a thyroid-associated gene product in detecting DTC cells in peripheral blood; however, Tg expression analysis produced ambiguous and diverse results (Ditkoff *et al*, 1996; Tallini *et al*, 1998; Ringel *et al*, 1999; Wingo *et al*, 1999; Bojunga *et al*, 2000; Bugalho *et al*, 2001; Takano *et al*, 2001; Eszlinger *et al*, 2002; Grammatopoulos *et al*, 2003). A recently published review concluded that Tg mRNA measurement is an invalid approach in the postoperative care of patients with DTC due to illegitimate expression of this marker in peripheral blood cells, although RT–PCR concepts in general are worthy of further investigations (Verburg *et al*, 2004). However, two other studies demonstrated results opposite to the conclusions drawn in this review, and reported a high specificity of Tg or TSH-R mRNA for thyroid cancer, hence promoting their application in DTC detection (Chinnappa *et al*, 2004; Li *et al*, 2004). Therefore, the experimental usefulness of Tg transcripts and the potential clinical relevance of this marker are still under debate.

A somewhat different method capable of detecting circulating thyroid carcinoma cells was first described by Ditkoff *et al* (1996). In their study, Tg mRNA was present in the peripheral blood of all patients with metastatic cancer, but absent in controls and in almost all individuals in disease remission. Investigators subsequently interpreted their observations to be correlated with extrathyroidal disease. Others reported a previously Tg mRNA negative control group revealing positive results following extended PCR amplification. The same was true for 10 human cell lines tested (Tallini *et al*, 1998). Healthy individuals who revealed circulating Tg mRNA transcripts in the peripheral blood were also described by Ringel *et al* (1998) using the RT–PCR technique. They interpreted their findings with a putative pool of circulating normal thyrocytes which produced Tg mRNA.

Refuting, we studied the peripheral blood of two patients lacking any residual thyroid tissue whatsoever because they underwent not only thyroidectomy for medullary thyroid carcinoma (originating from intrathyroidal C-cells and not from thyrocytes) but also – as an unusual additional treatment – radio-iodine therapy after surgery. The leukocyte fraction of these two individuals separated by density gradient centrifugation notedly revealed Tg mRNA expression (Figure 2). In addition, we have clearly demonstrated an ectopic transcription of Tg and TSH-R mRNA in all nucleated cell fractions of the peripheral blood using a highly sensitive two-step RT–PCR method (Figure 1). These findings strongly support the hypothesis that lymphocytes, monocytes and granulocytes express markers illegitimately, which were reported to be thyrocyte-specific. This phenomenon leads to a significant thyrocyte-independent PCR background signal, which interferes with assays using Tg and TSH-R mRNA. Based on these data, one can conclude that the hypothesis of circulating thyrocytes in healthy individuals as the source of peripheral Tg transcripts proposed by Ringel *et al* is improbable (Bojunga *et al*, 2000; Bugalho *et al*, 2001; Takano *et al*, 2001; Eszlinger *et al*, 2002). Experimental trials or future clinical applications dealing with Tg or TSH-R mRNA transcripts should be wary of false-positive results. Chelly *et al* (1989) reported that illegitimate transcription corresponds with a low expression level of spliced transcripts from specific genes in cells that are nonspecific for these transcripts. Due to identical promoter elements in specific and nonspecific cells, illegitimate or ectopic transcription is likely to be a result of a low promoter activity leading to nontranslated transcripts. The significance and widespread appearance of this phenomenon still need to be elucidated. A general tolerance of low-level promoter activity might be energetically advantageous for cellular metabolism rather than resting at complete promoter quiescence (Chelly *et al*, 1991).

Concluding, in detection of minimal residual disease in DTC, either time-consuming, sophisticated carcinoma cell harvesting or detection of circulating cancer cell via nonillegitimately transcribed sequences is feasible. In our opinion, the latter approach is preferable because it is more sensitive, specific, cost-effective and subject to routine laboratory automation.

As long as no thyrocyte-specific master genes are available, the amplification of minor genes not primarily involved in thyroid function but abnormally expressed in certain stages of tumorigenesis – due to gene deregulation and disturbances in molecular networks – is currently the only approach for detection of minimal residual disease in thyroid carcinoma.

In order to establish a single marker-based tumour cell detection assay, a consistently high expression of the chosen target mRNA sequence by the malignant tissue is required. Thus, we studied expression rates of the potential target sequences hTERT, CK 19, and onfFN in PTCs, FTCs, nonmalignant thyroid tissue, as well as in leukocyte subgroups. Our results demonstrate the complete absence of onfFN transcripts in each CD population associated with a high expression rate in DTC samples (Figures 1 and 3), suggesting that the onfFN transcript is a useful target sequence for thyroid carcinoma cell detection within the peripheral blood. Additionally, a significant difference in onfFN mRNA expression between PTCs and FTCs was shown (Figure 4), whereas the nonmalignant thyroid tissue samples lacked onfFN transcripts (data not shown). In further support of our results, Giannini *et al* (2003) assessed onfFN and galectin-3 mRNA in thyroid malignancies, and reported onfFN mRNA transcripts in almost 98% of PTCs.

Importantly, the analysis of peripheral blood from patients with known DTC metastatic disease revealed an onfFN mRNA expression in six out of nine patients identified by a well-defined cutoff value (Figure 5). This finding indicates the technical feasibility, reliability and potential clinical utility of this approach.

In summary, we have developed and optimised a specific, sensitive real-time RT–PCR assay using FRET technology in order to quantify absolute amounts of onfFN templates. A high expression rate of onfFN transcripts in DTCs was demonstrated, while onfFN mRNA was not found to be illegitimately transcribed by peripheral blood cells, but patients with DTC metastatic disease could be identified. These results may lead to a specific tool for monitoring micrometastases in the context of minimal residual disease or for assessing tumour response to therapy. The assay of onfFN-specific transcripts is a promising approach and worthy of further assessment in clinical trials.

## Figures and Tables

**Table 1:** Synopsis of the oligonucleotides used

**Sense primer**	**Sequence**	**Antisense primer**	**Sequence**	***T* (°C)**	**Bp**
PBGD-for	5′-attgctatgtccaccacagg-3′	PBGD-rev	5′-gcagggtttctagggtcttc-3′	59	253
Tg-for	5′-gccctctcttcagtggttgt-3′	Tg-rev	5′-ggaactccaaggaactggtc-3′	59	405
TSH-R-for	5′-tggggacagtgaagacatgg-3′	TSH-R-rev	5′-tgaagaaaccagccgtgttg-3′	58	332
CK19-for	5′-cagcattcacgggggctcc-3′	CK19-rev	5′-agcctgttccgtctcaaacttggt-3′	64	421
hTERT-for	5′-tgaacttgcggaagacagtg-3′	hTERT-rev	5′-gaggctgttcacctgcaaat-3′	57	290
onfFN-for	5′-tcttcatggaccagagatct-3′	onfFN-rev	5′-tatggtcttggcctatgcct-3′	56	215

## References

[bib1] Biscolla RP, Cerutti J, Maciel RM (2000) Detection of recurrent thyroid cancer by sensitive nested reverse transcription–polymerase chain reaction of thyroglobulin and sodium/iodide symporter messenger ribonucleic acid transcripts in peripheral blood. J Clin Endocrinol Metab 85: 3623–3627 11061512 10.1210/jcem.85.10.6876

[bib2] Bojunga J, Roddiger S, Stanisch M, Kusterer K, Kurek R, Renneberg H, Adams S, Lindhorst E, Usadel KH, Schumm-Draeger PM (2000) Molecular detection of thyroglobulin mRNA transcripts in peripheral blood of patients with thyroid disease by RT–PCR. Br J Cancer 82: 1650–1655 10817499 10.1054/bjoc.1999.1209 PMC2374521

[bib3] Bugalho MJ, Domingues RS, Pinto AC, Garrao A, Catarino AL, Ferreira T, Limbert E, Sobrinho L (2001) Detection of thyroglobulin mRNA transcripts in peripheral blood of individuals with and without thyroid glands: evidence for thyroglobulin expression by blood cells. Eur J Endocrinol 145: 409–413 11580997 10.1530/eje.0.1450409

[bib4] Burchill SA, Bradbury MF, Pittman K, Southgate J, Smith B, Selby P (1995) Detection of epithelial cancer cells in peripheral blood by reverse transcriptase–polymerase chain reaction. Br J Cancer 71: 278–281 7530983 10.1038/bjc.1995.56 PMC2033587

[bib5] Chelly J, Concordet JP, Kaplan JC, Kahn A (1989) Illegitimate transcription: transcription of any gene in any cell type. Proc Natl Acad Sci USA 86: 2617–2621 2495532 10.1073/pnas.86.8.2617 PMC286968

[bib6] Chelly J, Hugnot JP, Concordet JP, Kaplan JC, Kahn A (1991) Illegitimate (or ectopic) transcription proceeds through the usual promoters. Biochem Biophys Res Commun 178: 553–557 1650193 10.1016/0006-291x(91)90143-u

[bib7] Chinnappa P, Taguba L, Arciaga R, Faiman C, Siperstein A, Mehta AE, Reddy SK, Nasr C, Gupta MK (2004) Detection of thyrotropin-receptor messenger ribonucleic acid (mRNA) and thyroglobulin mRNA transcripts in peripheral blood of patients with thyroid disease: sensitive and specific markers for thyroid cancer. J Clin Endocrinol Metab 89: 3705–3709 15292293 10.1210/jc.2003-031967

[bib8] Datta YH, Adams PT, Drobyski WR, Ethier SP, Terry VH, Roth MS (1994) Sensitive detection of occult breast cancer by the reverse-transcriptase polymerase chain reaction. J Clin Oncol 12: 475–482 7509852 10.1200/JCO.1994.12.3.475

[bib9] de Kok JB, Zendman AJ, van de Locht LT, Ruers TJ, van Muijen GN, Mensink EJ, Swinkels DW (1999) Real-time hTERT quantification: a promising telomerase-associated tumor marker. Lab Invest 79: 911–912 10418832

[bib10] Ditkoff BA, Marvin MR, Yemul S, Shi YJ, Chabot J, Feind C, Lo Gerfo PL (1996) Detection of circulating thyroid cells in peripheral blood. Surgery 120: 959–964, (discussion 964–965) 8957481 10.1016/s0039-6060(96)80041-9

[bib11] Elisei R, Vivaldi A, Agate L, Molinaro E, Nencetti C, Grasso L, Pinchera A, Pacini F (2004) Low specificity of blood thyroglobulin messenger ribonucleic acid assay prevents its use in the follow-up of differentiated thyroid cancer patients. J Clin Endocrinol Metab 89: 33–39 14715824 10.1210/jc.2003-031341

[bib12] Eltahir EM, Mallinson DS, Birnie GD, Hagan C, George WD, Purushotham AD (1998) Putative markers for the detection of breast carcinoma cells in blood. Br J Cancer 77: 1203–1207 9579823 10.1038/bjc.1998.203 PMC2150149

[bib13] Eszlinger M, Neumann S, Otto L, Paschke R (2002) Thyroglobulin mRNA quantification in the peripheral blood is not a reliable marker for the follow-up of patients with differentiated thyroid cancer. Eur J Endocrinol 147: 575–582 12444888 10.1530/eje.0.1470575

[bib14] Giannini R, Faviana P, Cavinato T, Elisei R, Pacini F, Berti P, Fontanini G, Ugolini C, Camacci T, De Ieso K, Miccoli P, Pinchera A, Basolo F (2003) Galectin-3 and oncofetal-fibronectin expression in thyroid neoplasia as assessed by reverse transcription–polymerase chain reaction and immunochemistry in cytologic and pathologic specimens. Thyroid 13: 765–770 14558920 10.1089/105072503768499662

[bib15] Grammatopoulos D, Elliott Y, Smith SC, Brown I, Grieve RJ, Hillhouse EW, Levine MA, Ringel MD (2003) Measurement of thyroglobulin mRNA in peripheral blood as an adjunctive test for monitoring thyroid cancer. Mol Pathol 56: 162–166 12782763 10.1136/mp.56.3.162 PMC1187312

[bib16] Guller S, Ma Y, Raju U, Kadner S, Thung SF, Colasacco L, Malek A, Schneider H (2003) Release of oncofetal fibronectin from human placenta. Placenta 24: 843–850 13129681 10.1016/s0143-4004(03)00131-0

[bib17] Holt SE, Shay JW (1999) Role of telomerase in cellular proliferation and cancer. J Cell Physiol 180: 10–18 10362013 10.1002/(SICI)1097-4652(199907)180:1<10::AID-JCP2>3.0.CO;2-D

[bib18] Kim NW, Piatyszek MA, Prowse KR, Harley CB, West MD, Ho PL, Coviello GM, Wright WE, Weinrich SL, Shay JW (1994) Specific association of human telomerase activity with immortal cells and cancer. Science 266: 2011–2015 7605428 10.1126/science.7605428

[bib19] Li D, Butt A, Clarke S, Swaminathana R (2004) Real-time quantitative PCR measurement of thyroglobulin mRNA in peripheral blood of thyroid cancer patients and healthy subjects. Ann N Y Acad Sci 1022: 147–151 15251954 10.1196/annals.1318.024

[bib20] Lichtinghagen R, Diedrich-Glaubitz R, von Horsten B (1994) Identification of *Bordetella pertussis* in nasopharyngeal swabs using the polymerase chain reaction: evaluation of detection methods. Eur J Clin Chem Clin Biochem 32: 161–167 8031967 10.1515/cclm.1994.32.3.161

[bib21] Lichtinghagen R, Helmbrecht T, Arndt B, Boker KH (1995) Expression pattern of matrix metalloproteinases in human liver. Eur J Clin Chem Clin Biochem 33: 65–71 7632822 10.1515/cclm.1995.33.2.65

[bib22] Mazzaferri EL (2000) Long-term outcome of patients with differentiated thyroid carcinoma: effect of therapy. Endocr Pract 6: 469–476 11155222 10.4158/EP.6.6.469

[bib23] Musholt PB, Imkamp F, von Wasielewski R, Schmid KW, Musholt TJ (2003) RET rearrangements in archival oxyphilic thyroid tumors: new insights in tumorigenesis and classification of Hurthle cell carcinomas? Surgery 134: 881–889, (discussion 889) 14668719 10.1016/j.surg.2003.08.003

[bib24] Parker SL, Tong T, Bolden S, Wingo PA (1997) Cancer statistics, 1997. CA Cancer J Clin 47: 5–27 8996076 10.3322/canjclin.47.1.5

[bib25] Pedersen IB, Knudsen N, Jorgensen T, Perrild H, Ovesen L, Laurberg P (2003) Thyroid peroxidase and thyroglobulin autoantibodies in a large survey of populations with mild and moderate iodine deficiency. Clin Endocrinol (Oxf) 58: 36–42 12519410 10.1046/j.1365-2265.2003.01633.x

[bib26] Ringel MD, Balducci-Silano PL, Anderson JS, Spencer CA, Silverman J, Sparling YH, Francis GL, Burman KD, Wartofsky L, Ladenson PW, Levine MA, Tuttle RM (1999) Quantitative reverse transcription–polymerase chain reaction of circulating thyroglobulin messenger ribonucleic acid for monitoring patients with thyroid carcinoma. J Clin Endocrinol Metab 84: 4037–4042 10566646 10.1210/jcem.84.11.6164

[bib27] Ringel MD, Ladenson PW, Levine MA (1998) Molecular diagnosis of residual and recurrent thyroid cancer by amplification of thyroglobulin messenger ribonucleic acid in peripheral blood. J Clin Endocrinol Metab 83: 4435–4442 9851791 10.1210/jcem.83.12.5346

[bib28] Soria JC, Gauthier LR, Raymond E, Granotier C, Morat L, Armand JP, Boussin FD, Sabatier L (1999) Molecular detection of telomerase-positive circulating epithelial cells in metastatic breast cancer patients. Clin Cancer Res 5: 971–975 10353728

[bib29] Span PN, Sleegers MJ, van den Broek WJ, Ross HA, Nieuwlaat WA, Hermus AR, Sweep CG (2003) Quantitative detection of peripheral thyroglobulin mRNA has limited clinical value in the follow-up of thyroid cancer patients. Ann Clin Biochem 40: 94–99 12542917 10.1258/000456303321016231

[bib30] Spencer CA, Takeuchi M, Kazarosyan M, Wang CC, Guttler RB, Singer PA, Fatemi S, LoPresti JS, Nicoloff JT (1998) Serum thyroglobulin autoantibodies: prevalence, influence on serum thyroglobulin measurement, and prognostic significance in patients with differentiated thyroid carcinoma. J Clin Endocrinol Metab 83: 1121–1127 9543128 10.1210/jcem.83.4.4683

[bib31] Takano T, Matsuzuka F, Liu G, Miyauchi A, Yokozawa T, Kuma K, Amino N (1999) Analysis of splice variants of the fibronectin gene in thyroid carcinomas by reverse transcription–polymerase chain reaction: increased expression of oncofetal fibronectin mRNA in papillary carcinomas is not caused by the alternation in splicing. J Endocrinol Invest 22: 18–22 10090132 10.1007/BF03345473

[bib32] Takano T, Matsuzuka F, Sumizaki H, Kuma K, Amino N (1997) Rapid detection of specific messenger RNAs in thyroid carcinomas by reverse transcription–PCR with degenerate primers: specific expression of oncofetal fibronectin messenger RNA in papillary carcinoma. Cancer Res 57: 3792–3797 9288789

[bib33] Takano T, Miyauchi A, Matsuzuka F, Kuma K, Amino N (2000) Expression of oncofetal fibronectin messenger ribonucleic acid in fibroblasts in the thyroid: a possible cause of false positive results in molecular-based diagnosis of thyroid carcinomas. J Clin Endocrinol Metab 85: 765–768 10690888 10.1210/jcem.85.2.6344

[bib34] Takano T, Miyauchi A, Yoshida H, Hasegawa Y, Kuma K, Amino N (2001) Quantitative measurement of thyroglobulin mRNA in peripheral blood of patients after total thyroidectomy. Br J Cancer 85: 102–106 11437410 10.1054/bjoc.2001.1904 PMC2363919

[bib35] Tallini G, Ghossein R, Emanuel J, Gill J, Kinder B, Dimich AB, Costa J, Robbins R, Burrow GN, Rosai J (1998) Detection of thyroglobulin, thyroid peroxidase, and RET/PTC1 mRNA transcripts in the peripheral blood of patients with thyroid disease. J Clin Oncol 16: 1158–1166 9508203 10.1200/JCO.1998.16.3.1158

[bib36] Trummer A, Kadar J, Arseniev L, Petersen D, Ganser A, Lichtinghagen R (2000) Competitive cytokeratin 19 RT–PCR for quantification of breast cancer cells in blood cell suspensions. J Hematother Stem Cell Res 9: 275–284 10813542 10.1089/152581600319504

[bib37] Verburg FA, Lips CJ, Lentjes EG, de Klerk JM (2004) Detection of circulating Tg-mRNA in the follow-up of papillary and follicular thyroid cancer: how useful is it? Br J Cancer 91: 200–204 15213710 10.1038/sj.bjc.6601991 PMC2409974

[bib38] Weber T, Lacroix J, Weitz J, Amnan K, Magener A, Holting T, Klar E, Herfarth C, von Knebel Doeberitz M (2000) Expression of cytokeratin 20 in thyroid carcinomas and peripheral blood detected by reverse transcription polymerase chain reaction. Br J Cancer 82: 157–160 10638983 10.1054/bjoc.1999.0893 PMC2363210

[bib39] Wingo ST, Ringel MD, Anderson JS, Patel AD, Lukes YD, Djuh YY, Solomon B, Nicholson D, Balducci-Silano PL, Levine MA, Francis GL, Tuttle RM (1999) Quantitative reverse transcription–PCR measurement of thyroglobulin mRNA in peripheral blood of healthy subjects. Clin Chem 45: 785–789 10351986

